# The effect of tolvaptan on renal excretion of electrolytes and urea nitrogen in patients undergoing coronary artery bypass surgery

**DOI:** 10.1186/s12872-016-0341-0

**Published:** 2016-09-13

**Authors:** Tomoko S. Kato, Hiroshi Nakamura, Mai Murata, Kishio Kuroda, Hitoshi Suzuki, Yasutaka Yokoyama, Akie Shimada, Satoshi Matsushita, Taira Yamamoto, Atsushi Amano

**Affiliations:** 1 Department of Cardiovascular Surgery, Heart Center, Juntendo University, 2-1-1, Hongo, Bunkyo-ku, Tokyo 113-8421 Japan; 2 Division of Nephrology, Department of Internal Medicine, Juntendo University School of Medicine, Bunkyo-ku, Tokyo Japan

**Keywords:** Coronary artery bypass surgery, Tolvaptan, Renal excretion, Electrolyte, Urea nitrogen

## Abstract

**Background:**

Adequate fluid management is an important component of patient care following cardiac surgery. Our aim in this study was to determine the benefits of tolvaptan, an oral selective vasopressin-2 receptor antagonist that causes electrolyte-free water diuresis, in postoperative fluid management. We prospectively examined the effect of tolvaptan on renal excretion of electrolytes and urea nitrogen in cardiac surgery patients.

**Methods:**

Patients undergoing coronary artery bypass surgery were randomized to receive conventional loop diuretics (Group C, *n* = 30) or conventional loop diuretic therapy plus tolvaptan (Group T, *n* = 27). Fractional excretions of sodium (FENA), potassium (FEK) and urea nitrogen (FEUN) were measured in both groups during post-surgical hospitalization.

**Results:**

Urine output was greater with tolvaptan (Group T) than without it (Group C), and some patients in Group C required intravenous as well as oral loop diuretics. Serum sodium concentrations decreased after surgery in Group C, but were unchanged in Group T (postoperative day [POD] 3, 139.8 ± 3.5 vs. 142.3 ± 2.6 mEq/L, *p* = 0.006). However, postoperative FENA values in Group C did not decrease, and the values were similar in both groups. Serum potassium levels remained lower and FEK values remained higher than the preoperative values, but only in Group C (all *p* < 0.05). BUN increased postoperatively in both groups, but it remained higher than its preoperative value only in Group C (all *p* < 0.01). Group T showed an initial increase in BUN, which peaked and then returned to its preoperative value within a week. The FEUN increased postoperatively in both groups, but the change was more pronounced in Group T (POD7, 52.7 ± 9.3 vs. 58.2 ± 6.5 %, *p* = 0.025).

**Conclusions:**

Renal excretion of sodium and potassium reflects the changes in serum concentration in patients treated with tolvaptan. Patients treated only with loop diuretics showed a continuous excretion of sodium and potassium that led to electrolyte imbalance, whereas the combination of loop diuretics and tolvaptan increased renal urea nitrogen elimination. Tolvaptan therefore appears to be an effective diuretic that minimally affects serum electrolytes while adequately promoting the elimination of urea nitrogen from the kidneys in patients undergoing coronary artery bypass surgery.

**Trial registration:**

The present study is registered with the UMIN Clinical Trials Registry (ID: UMIN000011039)

## Background

Fluid management following cardiac surgery is one of the most important parts of postoperative cardiac patient care [1, 2]. Loop diuretics are commonly administrated postoperatively; however, their continuous usage sometimes leads to electrolyte imbalances, neurohumoral activation, worsening renal failure, and diuretic resistance [3]. Loop diuretics inhibit sodium reabsorption at the thick ascending limb of the loop of Henle and passively increase water excretion. Consequently, loop diuretics cause hyponatremia. However, the efficacy of loop diuretics is reduced under hyponatremia, resulting in a need to increase the dosage. This, in turn, further worsens the hyponatremia, creating a futile cycle [4]. In addition, loop diuretics increase sodium delivery to the distal segment of the distal tubule, which stimulates the aldosterone-sensitive sodium pump to increase sodium reabsorption in exchange for potassium. Volume depletion causes further increases in aldosterone secretion, resulting in excessive urinary potassium secretion and hypokalemia [3, 4]. Hypokalemia, in turn, may provoke both supraventricular and ventricular postoperative cardiac arrhythmias, with adverse effects on myocardial contractility [5]. These adverse effects of loop diuretics emphasize the need for better therapeutics for postoperative fluid management in cardiac patients.

One promising candidate is tolvaptan, an oral selective vasopressin V2-receptor antagonist that causes electrolyte-free water diuresis [6, 7]. Water balance in the early postoperative phase is modulated by the level of plasma arginine vasopressin (AVP), which increases in response to operative stress [8, 9]. AVP plays an important role in water reabsorption at the renal collecting duct and this increase causes an excessive body water imbalance during the early postoperative phase. The use of a vasopressin inhibitor like tolvaptan, which promotes water excretion without changes in renal hemodynamics or sodium and potassium excretion [6], therefore may represent an ideal strategy for maintaining fluid balance in patients undergoing cardiac surgery. Furthermore, tolvaptan may have benefits in reducing blood urea nitrogen (BUN) levels, which are associated with increased cardiovascular mortality and morbidity [10, 11]. Vasopressin promotes reabsorption of urea in the distal nephron, resulting in increased BUN [12]; therefore, vasopressin inhibition by tolvaptan treatment may also be effective for eliminating the excessive BUN associated with surgery-induced protein catabolism.

To the best of our knowledge, no detailed study has yet examined the effect of tolvaptan on renal electrolyte excretion following cardiac surgery. Therefore, we prospectively compared the fractional excretion of electrolytes and urea nitrogen in postoperative cardiac surgery patients following treatment with loop diuretics with or without tolvaptan.

## Methods

### Study design

Data on the effects of tolvaptan on changes in body weight and other clinical parameters after bypass surgery were collected at Juntendo University Hospital. Seventy patients undergoing off-pump coronary artery bypass surgery were prospectively enrolled in the study and randomized to either the group receiving conventional loop diuretic treatment only (Group C) or the group receiving conventional loop diuretic treatment plus tolvaptan (Group T). Patients in Group T received tolvaptan 7.5 mg once daily on postoperative days 1 and 2, in addition to conventional oral loop diuretics; intravenous loop diuretics were given only if needed. Tolvaptan was administered as needed from postoperative day 3 in Group T patients. Patients in Group C received only conventional loop diuretic therapy. Patients with renal insufficiency, such as those with chronic kidney disease stage 4 or greater, were excluded from the study. Patients requiring emergency surgery, those undergoing cardiopulmonary bypass, and those undergoing concomitant extracardiac/vascular and/or valvular surgery were also excluded. Laboratory values were serially measured after surgery for up to 7 days during hospitalization, and the fractional excretion of sodium (FENA), potassium (FEK), and urea nitrogen (FEUN) were calculated for both groups of patients.

The study was approved by the institutional review board of Juntendo University Hospital (UMIN000011039). All participants received detailed information about the study and provided written informed consent.

### Statistical analysis

Data are presented as mean ± SD. Normality was evaluated for each variable from normal distribution plots and histograms. Data obtained at the same time-point were compared between groups using Student’s unpaired two-tailed t-test for continuous variables and the chi-square test for categorical variables. Intragroup changes in preoperative vs. postoperative values were assessed with Student’s paired t-test. All data were analyzed using the Statistical Analysis Systems software JMP 11.0 (SAS Institute Inc., Cary, NC, USA).

## Results

### Patient characteristics

**Fig. 1:**
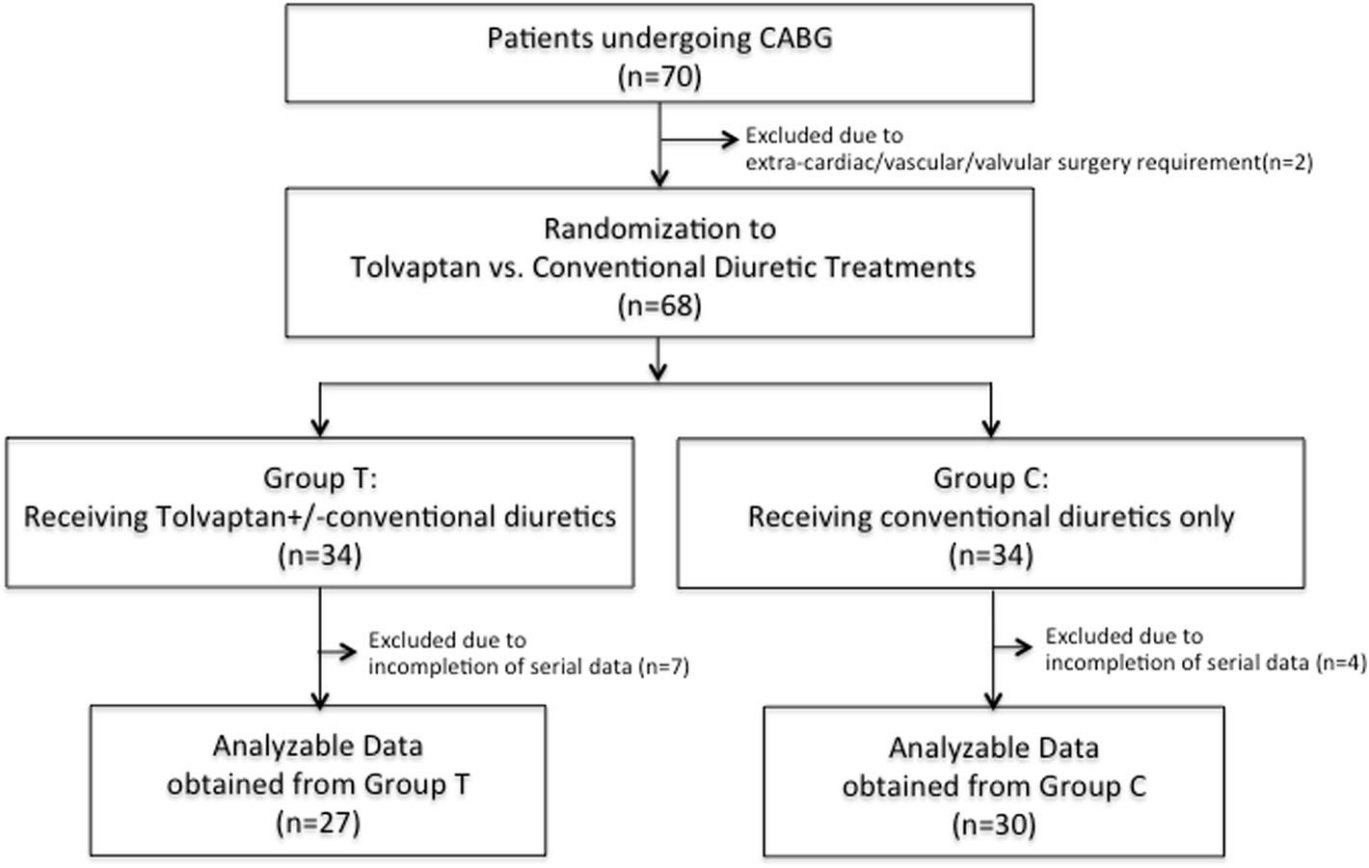
Flow chart of patient classification. A total of 70 patients scheduled to undergo coronary artery bypass surgery met the study inclusion criteria. Data for analysis were obtained from 30 patients treated with conventional diuretics only and from 27 patients treated with conventional diuretics plus tolvaptan

A total of 70 patients scheduled to undergo off-pump coronary artery bypass surgery were initially reviewed. Of those, 2 patients were excluded as they required another procedure in addition to bypass surgery. After randomization of patients into Groups T and C, incomplete datasets were collected from 7 patients in Group T and 4 patients in Group C. Therefore, analyzable data were obtained from 27 patients in Group T and 30 patients in Group C (Fig. 1).

Patient demographics, laboratory values prior to the surgery, and operative information for both groups are summarized in Table 1. Age, gender distribution, body surface area, and preoperative laboratory values, including the estimated glomerular filtration rate (eGFR), did not differ between the groups. The average operative duration was around 240 min, and the mean number of grafts was 3 in both groups.

**Table 1:** Patient characteristics

	Group T (*n* = 27)	Group C (*n* = 30)	*p* value
Age (years)	69.0 ± 10.7	70.1 ± 7.5	0.697
Men	23 (85.2 %)	20 (66.7 %)	0.189
Body surface area (m^2^)	1.70 ± 0.16	1.60 ± 0.16	0.061
Preoperative laboratory examinations			
Hb (g/dL)	11.9 ± 1.9	12.3 ± 1.2	0.341
Na (mEq/L)	141.5 ± 3.2	140.9 ± 3.1	0.537
K (mEq/L)	4.2 ± 0.2	4.3 ± 0.3	0.225
BUN (mg/dL)	14.3 ± 5.3	14.3 ± 5.5	0.965
Cre (mg/dL)	0.86 ± 0.32	0.80 ± 0.28	0.466
T-Bil (mg/dL)	1.0 ± 0.3	1.1 ± 0.2	0.065
TP (g/dL)	6.3 ± 0.5	6.3 ± 0.4	0.805
Alb (g/dL)	3.6 ± 0.3	3.4 ± 0.5	0.363
eGFR (ml/min./1.73 m^2^)	77.7 ± 31.0	76.4 ± 25.4	0.871
BNP (pg/mL)	263.1 ± 201.0	276.6 ± 197.5	0.814
Operative information			
Operative duration (minutes)	247.7 ± 54.3	240.1 ± 64.3	0.688
Number of grafts	3.0 ± 1.1	2.9 ± 1.3	0.899

Abbreviations not defined in the text: *Hb* hemoglobin, *Na* sodium, *K* potassium, *Cre* creatinine, *T-Bil* total bilirubin, *TP* total protein, *Alb* albumin, *BNP* brain natriuretic peptide

### Postoperative urinary output

**Fig. 2:**
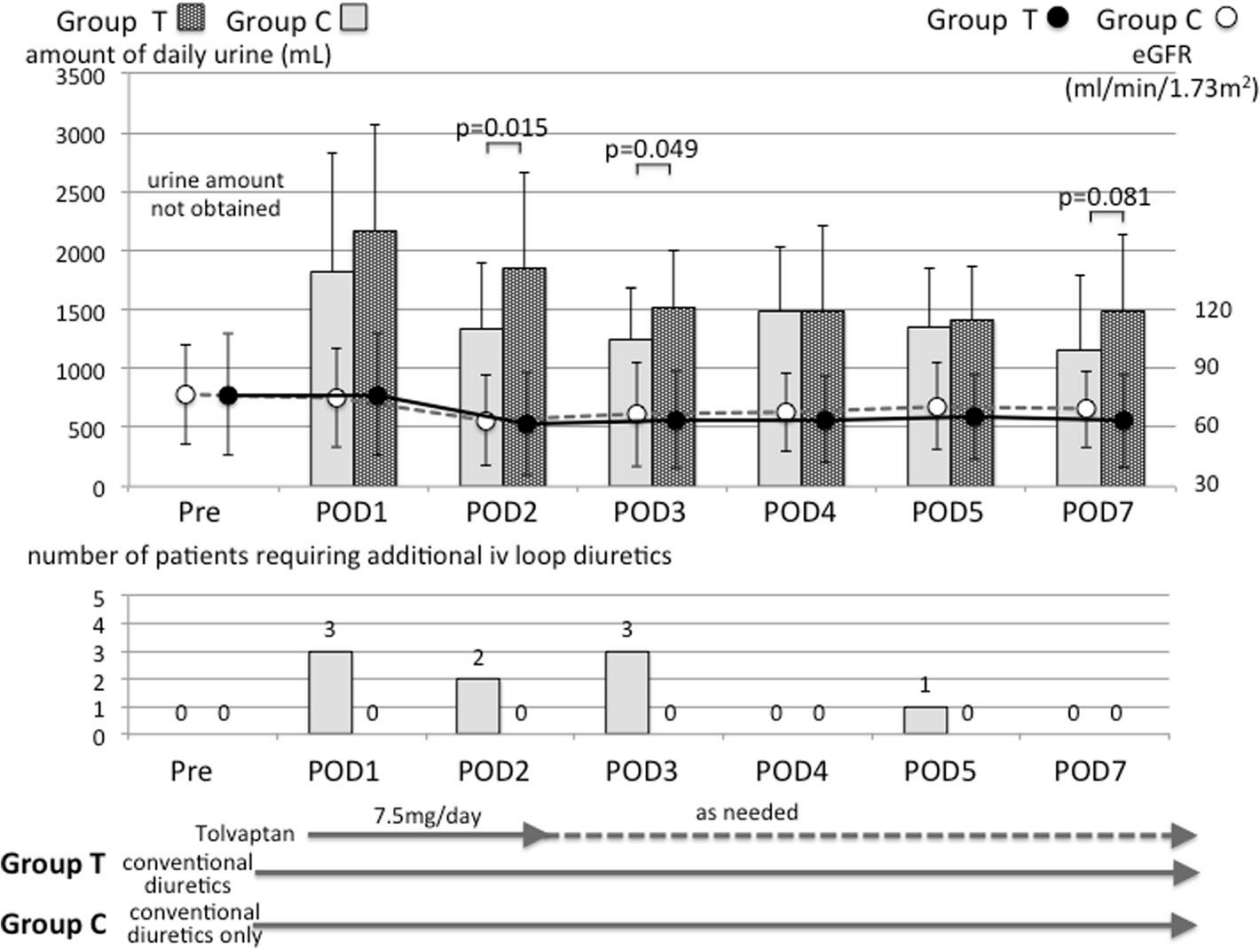
Serial changes in the amounts of daily urinary output after surgery and eGFR (*upper*), and numbers of patients who required intravenous loop diuretic administration (*lower*). Dark gray spotted bars and black circles indicate values for patients receiving conventional diuretic therapy plus tolvaptan (*Group T*) and gray bars and white circles indicate values for patients receiving conventional diuretic therapy only (*Group C*). POD, postoperative day

Both groups received loop diuretics after surgery in a routine manner in order to rectify postoperative hypervolemia. In our institution, Lasix® 40 mg per day orally is generally given to patients undergoing coronary bypass surgery. Additional Lasix® is administered intravenously if a patient’s hourly urine output is approximately less than 50 mL. In the present study, only some patients in Group C required additional intravenous loop diuretics (Fig. 2). Even so, the amounts of urine after surgery were greater in Group T than in Group C, and the differences were statistically significant on postoperative days 2 and 3. The estimated glomerular filtration rate (eGFR) values were not significantly different between the groups throughout the entire study period (Fig. 2).

### Serial changes in serum electrolytes, FENA, and FEK

**Fig. 3:**
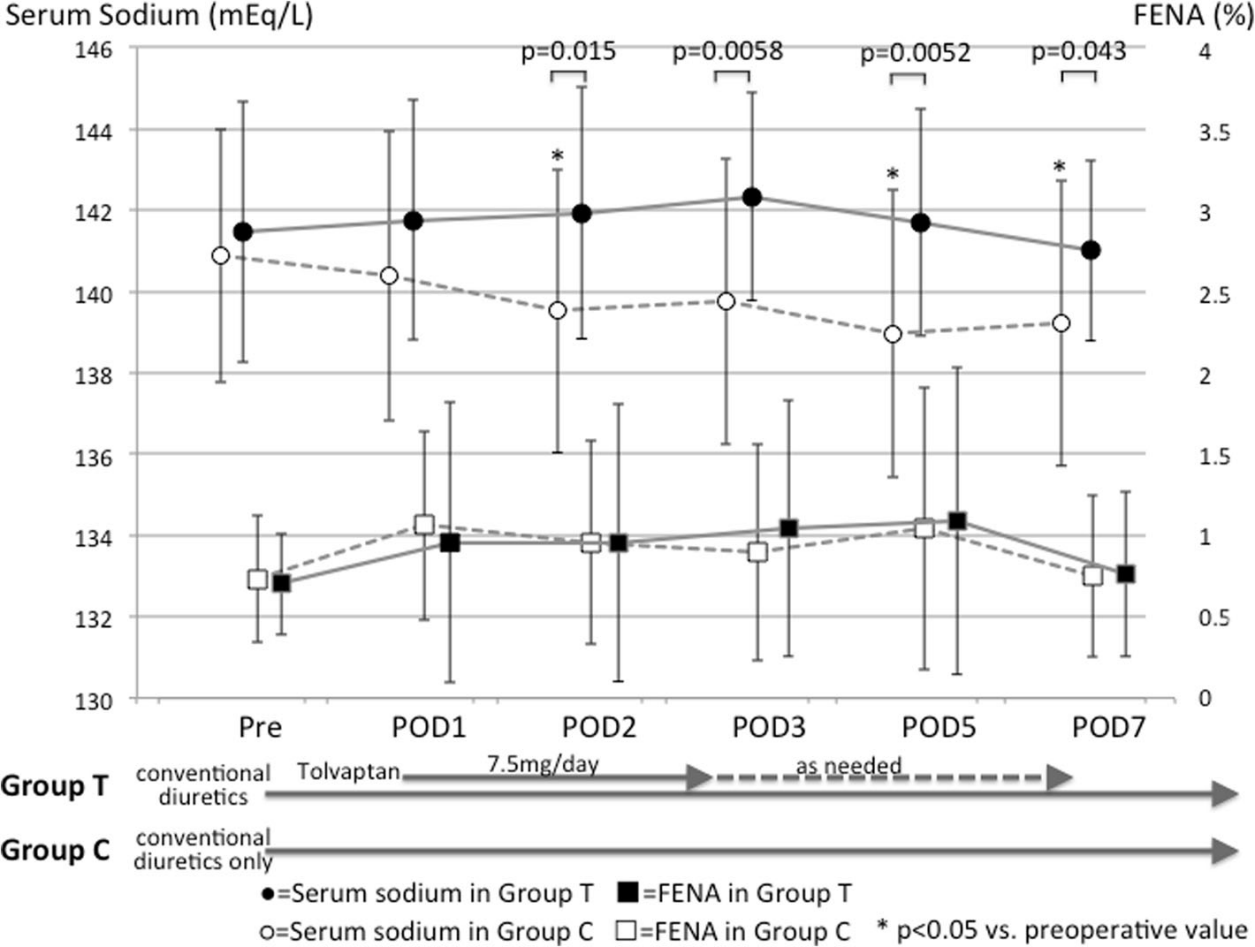
Serial changes in sodium concentrations (*upper*) and FENA (lower) in patients receiving conventional diuretics plus tolvaptan (*black symbols* and *solid lines*) or conventional diuretics only (*white symbols* and *dotted lines*). FENA, fractional excretion of sodium; POD, postoperative day

Figure 3 shows the serial changes in serum sodium concentrations and FENA before and after surgery in both groups. We were unable to obtain values at postoperative days 4 and 6 from some patients; therefore, the data from postoperative days 4 and 6 were omitted from the analysis. Serum sodium concentrations decreased postoperatively in Group C (from pre-surgery to postoperative days 2, 5, and 7, *p* = 0.046, 0.027, and 0.029, respectively), but they remained unchanged in Group T. Comparison of the values between the groups revealed that postoperative serum sodium concentrations at postoperative days 2, 3, 5, and 7 were higher in Group T than in Group C. However, even though the serum sodium concentrations decreased postoperatively in Group C, the renal sodium excretion in that group remained constant. In other words, FENA did not decrease in Group C.

**Fig. 4:**
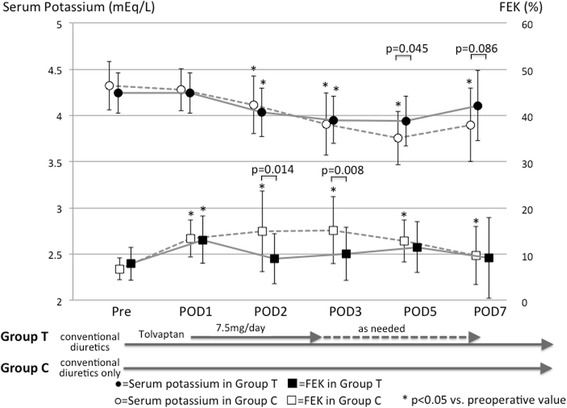
Serial changes in potassium concentrations (*upper*) and FEK (*lower*) in patients receiving conventional diuretics plus tolvaptan (*black symbols* and *solid lines*) or conventional diuretics only (*white symbols* and *dotted lines*). FEK, fractional excretion of potassium; POD, postoperative day

Figure 4 shows the serial changes in serum potassium concentrations and FEK before and after surgery. Serum potassium levels in both groups showed a gradual decrease after surgery until postoperative day 3 (from pre-surgery to postoperative day 3: for 4.2 ± 0.2 to 3.9 ± 0.2 mEq/L for Group T and 4.3 ± 0.3 to 3.9 ± 0.3 mEq/L for Group C, both *p* < 0.001). After postoperative day 3, serum potassium levels in Group T returned to the preoperative range, whereas the levels stayed lower in Group C. The FEK values increased from their preoperative value on postoperative day 1 in both groups (both *p* < 0.001). In Group C, the FEK values further increased until postoperative day 3, and they remained higher than the preoperative value (from pre-surgery to postoperative days 2, 3, and 5, all *p* < 0.001). In Group T, the FEK value, which was higher on postoperative day 1 than on the preoperative value, decreased and returned to the preoperative range after postoperative day 2.

### Serial changes in blood urea nitrogen (BUN) and FEUN

**Fig. 5:**
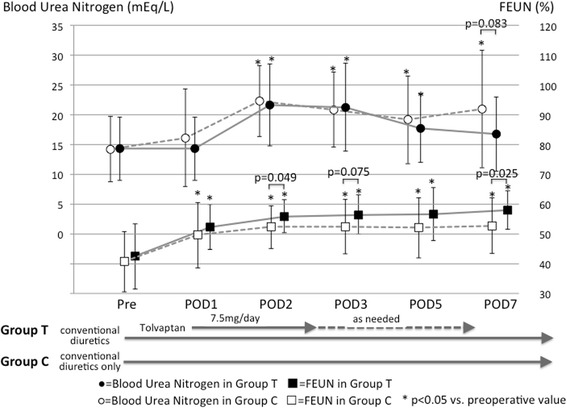
Serial changes in BUN levels (*upper*) and FEUN (*lower*) in patients receiving conventional diuretics plus tolvaptan (*black symbols* and *solid lines*) or conventional diuretics only (*white symbols* and *dotted lines*). FEUN, fractional excretion of urea nitrogen; POD, postoperative day

Figure 5 shows the serial changes in BUN levels and FEUN before and after surgery. BUN increased postoperatively in both groups, but remained higher than its preoperative value until postoperative day 7 in Group C (from pre-surgery to postoperative day 7, 14.3 ± 5.5 to 21.0 ± 9.9 mg/dL, *p* = 0.0003). In Group T, the increase in BUN peaked on postoperative day 2 and then gradually decreased to its preoperative value.

In both groups, FEUN increased postoperatively and remained higher than the preoperative values. The increase in postoperative FEUN was more pronounced in Group T. Intergroup comparison showed that the FEUN values at postoperative days 2 and 7 were statistically higher in Group T than in Group C (*p* = 0.049 and 0.025, respectively).

### Postoperative clinical courses

The postoperative clinical courses in all patients are compared in Table 2. Because some patients were seen at outside community hospitals after discharge, we were unable to gather long-term data, such as laboratory examinations, from all patients. No differences were evident in mortality between the groups up to 2.9 years after the surgery (mean 2.1 years). The occurrence of perioperative adverse events was not statistically different between the groups. None of the patients required renal replacement therapy after the surgery; however, one patient in Group T (3.7 %) and 4 in Group C (13 %) developed RIFLE classification-R (risk) acute kidney injury (AKI) [13].

**Table 2:** Postoperative clinical course

	Group T (*n* = 27)	Group C (*n* = 30)	*p* value
Mortality, *n* (%)^a^	0 (0 %)	0 (0 %)	-
Duration of intensive care unit stay (days)	1.3 ± 1.9	1.2 ± 1.3	0.816
Bleeding amount (mL)	324.2 ± 273.4	308.4 ± 202.3	0.804
Intubation time (hours)	9.3 ± 5.3	11.2 ± 6.4	0.230
Perioperative adverse events, *n* (%)			
Neurological complications, *n* (%)	0 (0 %)	0 (0 %)	-
Arrhythmia, *n* (%)	6 (22.2 %)	8 (26.7 %)	0.697
Renal replacement therapy, *n* (%)	0 (0 %)	0 (0 %)	-
RIFLE classification-Risk, *n* (%)	1 (3.7 %)	4 (13.3 %)	0.467

^a^The observation period for mortality was 2.9 years (mean 2.1 years)

## Discussion

In the present investigation, both groups of patients received loop diuretics after surgery; therefore, the data did not reflect any independent effect of tolvaptan on renal excretion of electrolytes and urea nitrogen, as these were also influenced by the conventional loop diuretics. Even so, we believe that our detailed observations of the serial daily changes in laboratory values in serum and urine can aid in understanding how tolvaptan affects renal excretion mechanisms in patients undergoing cardiac surgery.

Our data show that (i) the amount of daily urine output was well maintained and somewhat greater with tolvaptan (Group T) than without it (Group C), although some patients in Group C required intravenous loop diuretic administration; (ii) serum sodium concentrations decreased after surgery only in Group C, but the postoperative FENA values were not significantly different with or without tolvaptan; (iii) in the absence of tolvaptan (Group C), postoperative serum potassium concentrations decreased and stayed lower than the preoperative level, with an associated increase in FEK. By contrast, the addition of tolvaptan (Group T) maintained the postoperative serum potassium levels at essentially the preoperative levels; and (iv) both groups showed an increase in BUN and FEUN after surgery, but tolvaptan treatment led to a higher FEUN and a rapid decrease in BUN as time progressed.

We speculate that the similar FENA values with and without tolvaptan after surgery may reflect an enhancement of the effectiveness of loop diuretics by tolvaptan. Previous papers have also noted that concomitant administration of tolvaptan enhances the effects of loop diuretics [14]. Nishizaki et al. showed an enhancement of natriuresis that could be an indirect effect of tolvaptan, and they assumed that tolvaptan relieves renal congestion, thereby enhancing the performance of the loop diuretics [14]. Furthermore, the dose-response curves for the loop diuretics [15] indicate that patients with renal insufficiency and/or heart failure require a higher diuretic dose to achieve the desired FENA. Therefore, our finding that the patients in Group T showed a similar FENA value to patients in Group C under a smaller loop diuretic dosage could be confirmation that tolvaptan enhances loop diuretic-induced natriuresis.

In the current study, both groups of patients were placed on a salt-restricted diet soon after surgery, and the amount of peroral sodium intake did not likely differ between the groups. Therefore, the higher serum sodium concentration seen in Group T, compared to Group C, might have resulted from the effective free-water diuresis induced by tolvaptan in Group T. Indeed, the amount of daily urine volume was larger in Group T soon after surgery (Fig. 2). In addition, the serum albumin levels were substantially higher in Group T than Group C after surgery (e.g., postoperative day 5, 3.3 ± 0.3 vs. 3.5 ± 0.2 mg/dL, *p* = 0.024). Probably because the number of studied patients was too small, we failed to show a tolvaptan effect on eGFR values (Fig. 2); however, there was a tendency for the patients in Group C to develop RIFLE classification-R (risk) AKI more frequently than the patients in Group T. This result may reflect the findings by Shirakabe et al., who reported that early administration of tolvaptan could prevent exacerbation of AKI in heart failure patients [16].

The decrease in serum potassium concentrations and the slight increase in FEK after surgery in Group C are reasonable responses to loop diuretic therapy. Group T patients also received loop diuretics, although the dosage was smaller than in Group C, and the changes in their serum potassium and FEK values soon after surgery showed similar trends to those in Group C.

The increase in BUN just after surgery most likely reflects surgery-induced protein catabolism. Although the operative duration was about 4 h and the peri-operative fasting period was approximately 1 day in both groups, maintenance fluid replacement, together with the fasting, would elicit protein degradation. However, the sustainment of this increase in BUN after surgery may also reflect neurohormonal activation and renal hypoperfusion [10, 11, 17, 18]. Testani et al. reported that excessive neurohormonal activation, as estimated by BUN elevation, can predict potential adverse effects of loop diuretics [19]. Several studies have suggested that BUN is a simple and significant prognostic marker associated with morbidity and mortality in patients with heart failure [10, 11, 17, 18, 20], and increases in BUN appear to be associated with cardiovascular mortality [11, 20]. In patients with heart failure, an increase in renin-angiotensin aldosterone system activity provokes urea reabsorption at the proximal tubule, while sympathetic nervous system activation promotes urea reabsorption at the distal and proximal tubules, and elevation of AVP upregulates urea transporters in the collecting tubule [12, 18]. Our observations indicate that tolvaptan could reasonably modulate this AVP-associated urea reabsorption, as indicated by the decrease in BUN in Group T.

Operative stress induces neurohormonal activation and increases serum AVP levels [8, 9]. The measurements of serum AVP levels in 239 critically ill patients and 70 healthy volunteers by Jochberger et al. confirmed higher AVP concentrations in patients undergoing cardiac surgery than in patients undergoing noncardiac surgery or who had nonsurgical diseases [21]. Terazawa et al. reported that serum AVP levels increased during and soon after cardiac surgery, and that the increase was larger in patients with more severe conditions [22]. Therefore, patients undergoing cardiac surgery could have a volume overload that could be related to increased plasma AVP due to operative stress. We believe that tolvaptan may be an ideal diuretic for neurohormonal control as well as maintenance of adequate electrolyte balance. Furthermore, tolvaptan may cause excretion of the excessive BUN produced by protein catabolism and operative stress, which may be beneficial for long-term prognoses.

We admit that tolvaptan is a relatively expensive medication; however, it effectively enables diuresis without causing electrolyte imbalance, which is helpful in the management of postsurgical patients who are at high risk for cardiac arrhythmia and for progression to acute kidney injury. Therefore, the high cost of this treatment may be offset by reductions in the lengths of stays in the intensive care unit and further costly postsurgical treatment. Additional studies are needed to analyze the efficacy of tolvaptan on a medical-care cost basis in patients undergoing cardiac surgery. Moreover, elevated BUN and low sodium concentration have well known associations with poor patient prognosis [10, 11, 16], and tolvaptan treatment is expected to improve survival in heart failure patients by normalization of these factors [23]. The present observation that urea nitrogen excretion was enhanced by tolvaptan treatment without affecting sodium concentration supports this expectation. The effect of tolvaptan on long-term prognosis in patients undergoing cardiac surgery should be further investigated.

The present study had several limitations. First, this was a single-center, retrospective, observational analysis with a small number of patients. According to the protocol, tolvaptan was given only on postoperative days 1 and 2 in Group T, and the decision to continue tolvaptan was up to the attending doctor. Therefore, among the Group T patients, some received tolvaptan throughout their hospital stay and some received it for only 2 days. This may have affected the data obtained after postoperative day 3, which complicates the interpretation of our results. In addition, because most of the patients were able to consume fluids orally within a day after surgery, we were unable to estimate the effect of intravenous fluid administration on serum and/or urinary electrolytes. We also did not measure plasma AVP levels or urine Aquaporin-2, which have been reported to be predictive markers of the response to tolvaptan [24, 25]. Other than diuretics, we did not include information on the use of other drugs, such as inotropic agents and beta-blockers. Furthermore, we reviewed only the short-term clinical course and did not review long-term outcomes. Lastly, the objective variables had a relatively larger standard deviation than we initially expected, so the statistical power derived from the present results was not sufficient to draw conclusions. However, this is a preliminary analysis and we would like to enroll greater numbers of patients in more specific studies.

## Conclusions

In conclusion, loop diuretics can cause continuous excretion of sodium and potassium, irrespective of their serum concentrations, which may result in electrolyte imbalance. The addition of tolvaptan in the present study prevented the generation of an electrolyte imbalance in the cardiac surgery patients investigated here. Furthermore, tolvaptan seems to enhance the effectiveness of loop diuretics. Tolvaptan also showed potential to promote the excretion of urea nitrogen produced by protein catabolism and in response to operative stress, and it does so more effectively than loop diuretics in patients undergoing coronary artery bypass surgery.

## Abbreviations

AVP: Arginine vasopressin
BUN: Blood urea nitrogen
eGFR: Estimated glomerular filtration rate
FEK: Fractional excretion of potassium
FENA: Fractional excretion of sodium
FEUN: Fractional excretion of urea nitrogen
POD: Postoperative day
